# Exploring the role of negative urgency in body dissatisfaction in young adults

**DOI:** 10.1177/13591053261430272

**Published:** 2026-03-13

**Authors:** Janette Watkins, Emma Herms, Emma Schiestl, Krista Wisner

**Affiliations:** 1 Pennsylvania State University, University Park, PA, USA; 2 Indiana University, Bloomington, IN, USA

**Keywords:** emotion regulation, impulsivity, sex differences, mental health, body image

## Abstract

Body dissatisfaction is a key predictor of poor mental health and disordered eating, yet prior studies often examine its psychological correlates in isolation or within narrow demographic groups. This study tested whether negative urgency—the tendency to act impulsively in response to negative emotions—mediates the association between depression and body dissatisfaction in young adults. Participants were drawn from the Nathan Kline Institute Rockland Sample (*N* = 232; 127 males, 105 females). A composite body dissatisfaction score was derived from Shape Concern and Weight Concern subscales. Path analyses indicated that negative urgency mediated the depression–body dissatisfaction link in the full sample, driven by significant mediation among males but not females. Specificity analyses for Shape and Weight Concern showed the same pattern. Findings highlight negative urgency as a vulnerability factor connecting mood symptoms to body dissatisfaction, underscoring sex differences and the need for targeted interventions addressing impulsivity and emotion regulation.

## Introduction

Body dissatisfaction, a critical aspect of self-image, is closely linked to maladaptive behaviors such as disordered eating and social withdrawal (Merino et al., 2024; Neumark-Sztainer et al., 2006). There also appear to be important associations between body dissatisfaction and mental health factors such as anxiety, depression, and impulsivity (Kostanski and Gullone, 1998; Pietrabissa et al., 2020), which can disrupt daily functioning and diminish overall well-being (Paxton et al., 2006, 2022). These associations are suggested to differ across males and females (Neves et al., 2017). Concerningly, the associations between body dissatisfaction and mental health factors are often studied in isolation (e.g. just depression, or anxiety, or impulsivity considered), and often without regard to sex differences. Given the co-occurring nature of these dimensions, a more integrative approach may provide necessary clarifications to refine targeted interventions aimed to increase body satisfaction across groups (Cash and Smolak, 2011).

Biological sex plays a crucial role in shaping body image, with women consistently reporting higher body dissatisfaction than men. For example, a recent review found women experienced more dissatisfaction for their physical appearance, which was associated with poor outcomes such as reduced physical activity levels and lower well-being (Merino et al., 2024). Biological sex differences in body dissatisfaction may stem from lifelong exposure to societal ideals that emphasize thinness and attractiveness for women, compared to less stringent appearance standards for men (McCabe and Ricciardelli, 2004). For women, body dissatisfaction appears rooted in weight, shape, and aging-related changes, often exacerbated by media and cultural expectations (Karazsia et al., 2017; McCreary and Sasse, 2000). For men, concerns are more often centered around muscularity and body size, reflecting internalized pressures to appear strong and fit, rather than thin. Such societal pressures contribute to a stronger link between body dissatisfaction and mental health outcomes in women than men, which suggests a need for individualized interventions that promote positive body image and healthy behaviors in adults (Tiggemann and Lynch, 2001).

Negative urgency—a facet of impulsivity defined as the tendency to act rashly when experiencing negative emotions—has emerged as a key psychological mechanism linking emotional distress to body dissatisfaction. This construct is one of five impulsivity traits on the UPPS-P scale (Cyders et al., 2014; Whiteside and Lynam, 2001). Among the UPPS-P measures, negative urgency consistently shows the strongest associations with disordered eating and body-related distress (Fischer et al., 2008), especially in the presence of body shame, suggesting that impulsivity may amplify the impact of body concerns on maladaptive coping strategies (Cyders and Smith, 2008; Settles et al., 2012). Longitudinal findings also support the temporal relationship between trait-level impulsivity and the development of disordered eating patterns (Stice and Shaw, 2002). Taken together, these findings highlight negative urgency as a theoretically and empirically supported mediator between negative emotional states (e.g. depression) and body dissatisfaction. That is, while mental health symptoms like anxiety and depression are related to body dissatisfaction, they may operate through negative urgency, rather than independently explaining body dissatisfaction.

State anxiety—the acute emotional response of fear or worry in reaction to stressors—also plays an important role in body dissatisfaction, especially in social evaluative contexts (Vannucci and Ohannessian, 2018). Elevated state anxiety has been shown to intensify appearance-related concerns and self-focused attention, which leads to negative body perceptions (Ohannessian et al., 1999; Vannucci and Ohannessian, 2018). General anxiety has also been associated with increased social appearance anxiety and disordered eating, even after accounting for depression and perfectionism (Levinson and Rodebaugh, 2015). Theoretical models suggest anxiety can prompt self-critical beliefs and avoidance behaviors, which increase vulnerability to depression (Jacobson and Newman, 2014).

State depression—characterized by present feelings of sadness, low pleasure, worthlessness, or guilt—is another critical factor influencing body dissatisfaction. Depressive symptoms may worsen body dissatisfaction by reducing self-esteem, fueling negative self-evaluation, and prompting rumination on perceived flaws (Joiner et al., 1997; Nolen-Hoeksema, 1991). Targeting anxiety and depression through evidence-based treatments such as cognitive-behavioral or mindfulness-based interventions may improve body satisfaction and reduce psychopathology risk (Amirshahi et al., 2024). However, a significant proportion of individuals do not experience meaningful improvement, prompting researchers to examine intermediary mechanisms like negative urgency that may explain individual variability in treatment response (Goldschmidt et al., 2025).

The current study investigates the role of negative urgency in body dissatisfaction, while modeling the contributions of state depression, state anxiety, BMI, age, and biological sex. We hypothesize that negative urgency will not only contribute unique variance in the prediction of body dissatisfaction, but importantly that negative urgency will mediate the relationship between depression and body dissatisfaction. The present study prioritizes depression as the primary predictor because depressive symptoms show stronger and more consistent links with distress-driven impulsivity (negative urgency) than anxiety, which was therefore modeled as a covariate to isolate this pathway (Moeller et al., 2001; Swann et al., 2008). The overall objective of the current study is to refine existing models of body dissatisfaction by identifying a specific cognitive-affective pathway through which depression—via increased susceptibility to emotional distress—may lead to worsen body dissatisfaction. Given existing literature on sex-related differences in body dissatisfaction, we subsequently explore if the relationships between depression, negative urgency, and body dissatisfaction generalize across subgroups based on biological sex. Ultimately, this research seeks to clarify key psychological and demographic factors associated with body dissatisfaction, to inform development of targeted and tailored interventions to enhance well-being and reduce the comorbid risks associated with body dissatisfaction.

## Methods

### Participants

The study utilized publicly available data from the Nathan Kline Institute (NKI) Rockland Sample, a large community-based dataset designed for investigating neurocognitive and mental health variables across the lifespan, with details published elsewhere (Nooner et al., 2012). Access and procedures with the dataset were approved by the Institutional Review Board (Protocol #2009962152). For the current study, we focused on young adult participants aged 18–30 years old to examine variables associated with body dissatisfaction during this critical developmental period. After merging the psychological and demographic variables described below into an initial dataset of unique participants, the sample was observed to be predominately female. To balance representation of males and females across the age range, optimal matching was performed. This approach aimed to reduce age-related confounding while improving representation across ages, rather than achieving equal sample sizes by sex. The resulting final sample consisted of 232 participants (*n* = 127 Males; *n* = 105 Females). Key demographics characterizing the sample can be found in Table 1.

**Table 1.** Demographics of young adult participants.

Demographic characteristic	Category	*n*	Percentage or mean
Biological sex	Male	127	54.7%
	Female	105	45.3%
Age (Mean ± SD)	Males	–	22.62 ± 3.79
	Females	–	22.65 ± 3.42
Race	Male White	97	76.37%
	Male Non-White	30	23.62%
	Female White	71	67.61%
	Female Non-White	34	32.38%

### Measures

The *primary dependent variable of interest, body dissatisfaction*, was measured by the Eating Disorder Examination Questionnaire (EDEQ; Aardoom et al., 2012). A composite score was created by summing two distinct subscales from the EDEQ: Shape Concern (eight items) and Weight Concern (five items). The Shape Concern subscale assesses broader and more subjective dissatisfaction with one’s body, including discomfort with body shape, feeling fat, and distress about specific body areas. In contrast, the Weight Concern subscale focuses more narrowly on preoccupations with body weight, fear of weight gain, and desires to lose weight. Specifically, the composite score was calculated by summing the mean scores of the Shape Concern and Weight Concern subscales, wherein each was initially derived by averaging responses from their respective items. This composite was used to capture global body dissatisfaction while reducing model complexity; subscale-specific analyses were conducted to assess potential sex-specific distinctions. While the Shape Concern and Weight Concern subscales are moderately to highly correlated (typically *r* = 0.70–0.85 in clinical and non-clinical samples (Luce and Crowther, 1999)), they are theorized to reflect overlapping but distinct aspects of body dissatisfaction. To assess if diverging patterns exist, supplemental analyses examined Shape Concern and Weight Concern subscales independently. The remaining two subscales, “Eating Concern” and “Restraint,” were not analyzed as they focus on eating-related behaviors rather than body dissatisfaction itself. The EDEQ response scale ranges from 0 (no concern) to 6 (extreme concern), and the questionnaire asks respondents to consider their experiences over the past 28 days as a relative state measure. The EDEQ has been validated in community (Peláez-Fernández et al., 2012) and diagnostic (Aardoom et al., 2012) samples.

The *key mediating independent variable of interest, negative urgency*, was measured by the UPPS-P Impulsive Behavior Scale (UPPS; Whiteside and Lynam, 2001), specifically using the “Negative Urgency” subscale (13 items), which reflects a tendency to act impulsively when experiencing strong negative emotions. The UPPS includes other subscales not examined in our analyses (i.e. lack of premeditation, lack of perseverance, sensation seeking, and positive urgency). Using the UPPS, respondents rate their general tendencies on a scale from 1 (strongly disagree) to 4 (strongly agree). The UPPS has been validated in community (D’Acremont and Linden, 2005) and diagnostic (Dugré et al., 2019) samples. The UPPS has also been shown to have measurement invariance across age and sex groups in adults (Argyriou et al., 2020).

The *foundational independent variable of interest, depression*, was measured using the Beck Depression Inventory-II (BDI). The BDI measures the severity of depressive symptoms across cognitive, affective, and physical manifestations over the past 2 weeks using 21 items. The Likert scale for the BDI ranges from 0 (No or minimal presence of the symptom) to 3 (Severe presence of the symptom). The BDI total score was employed in analyses. The BDI has been validated in general and hospital adult populations (García-Batista et al., 2018).

Additional covariates included in all analyses were age, Body Mass Index (BMI) calculated based on measurements of weight and height, plus a measure of state anxiety as it often co-occurs with body dissatisfaction and depression. The State-Trait Anxiety Inventory (STAI) measures both recent (state) and general tendencies (trait) levels of anxiety using 20 parallel items for each dimension. The Likert scale for STAI ranges from 1 (not at all) to 4 (very much). The STAI state anxiety total score was employed in analyses.

### Statistical analysis

All data analysis was completed in R version (2024.09.1). Continuous variables were residualized by race (White vs non-White) to reduce confounding due to unequal racial representation and subgroup imbalance. This approach allowed us to retain race-related variance control without unstable stratified models; however, findings should be interpreted within the context of a predominantly White sample. This was conducted due to the poor representation of non-White participants in the sample and unequal distribution of non-White participants in subgroup analyses, combined with prior evidence of race relationships with body dissatisfaction (Gillen and Lefkowitz, 2012; Olson et al., 2020). Additionally, prior to analyses, normalization procedures were applied to continuous variables using the BestNorm package to improve distributions where necessary. Shapiro-Wilk tests were conducted to confirm improved normality in the transformed data. Scaled (demeaned) scores were used for all continuous variables in analyses to standardize regression coefficients, while a binary variable coded biological sex. Biological sex is used here to reflect sex assigned at birth in the available dataset; gender identity and gendered experiences were not assessed and therefore cannot be inferred.

Major analyses proceeded in four stages. First, path models tested the hypothesized mediation in the full young adult sample; specifically, this examined whether negative urgency mediates the relationship between depression and the body dissatisfaction composite when including the above specified covariates. Second, exploratory path models were conducted within each biological sex subgroup to evaluate potential sex differences in mediation pathways using the body dissatisfaction composite as the outcome. Third, supplemental analyses assessed subscale specificity for body dissatisfaction; using Shape Concern and Weight Concern as separate outcomes, mediation models were tested within males and females separately. In all models, mediation effects were tested using bias-corrected bootstrap confidence intervals. Interactions of negative urgency and biological sex were not examined, as relationships between negative urgency and body dissatisfaction have not been thoroughly tested in equally powered subgroups to support a priori predicted moderation effects (Cyders, 2013; Fischer et al., 2013; Steward et al., 2017).

## Results

### Primary analysis: Composite body dissatisfaction mediation

Using the entire young adult sample (*N* = 232), a path analysis was conducted to test whether negative urgency mediated the relationship between depression and the body dissatisfaction composite (sum of Shape Concern and Weight Concern), while controlling for the effects of BMI, biological sex, age, and state anxiety (Figure 1). The combined sample analysis revealed significant mediation effects. Depression had a significant positive association with negative urgency (*a* = 0.466, *p* < 0.01) and negative urgency, in turn, predicted higher body dissatisfaction (*b* = 0.121, *p* < 0.05). The direct effect of depression on body dissatisfaction remained significant, indicating partial mediation (*c*’ = 0.266, *p* < 0.01). The model explained 24.3% of the variance in body dissatisfaction (*p* = 0.034), indicating a moderate effect size for psychosocial predictors. Among the covariates, biological sex was a significant predictor, with females showing higher body dissatisfaction than males (*p* < 0.001). BMI was also a significant covariate (*p* < 0.001).

**Figure 1.**
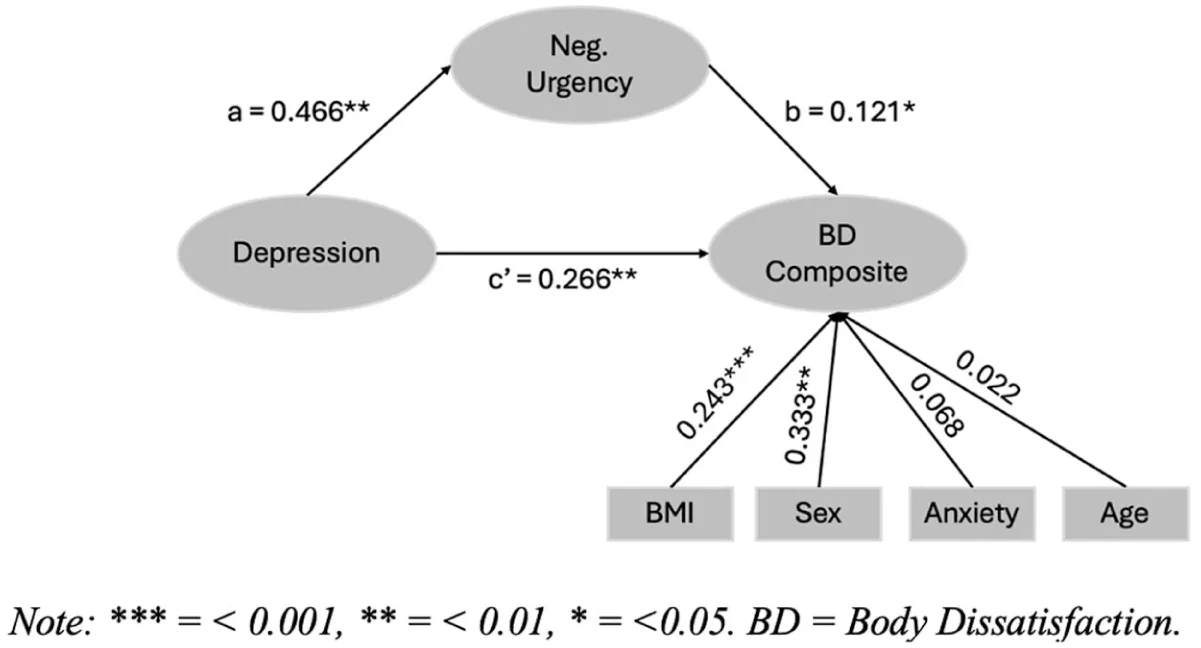
Composite meditation model in full sample.

### Exploratory sex difference analysis

To explore potential sex differences in the mediation model, separate path analyses were conducted for males and females. Among males (*n* = 127), the mediation model revealed significant effects (Figure 2, Panel A). Depression was significantly associated with negative urgency (*a* = 0.377, *p* < 0.001), and negative urgency significantly predicted body dissatisfaction (*b* = 0.156, *p* < 0.05). The direct effect of depression on body dissatisfaction also remained significant (*c*’ = 0.288, *p* < 0.001), indicating partial mediation. The model accounted for 16.98% of the variance in body dissatisfaction (*p* = 0.042), representing a modest but meaningful effect size for psychosocial predictors, with significant covariates of age (*p* < 0.05) and BMI (*p* < 0.001).

**Figure 2.**
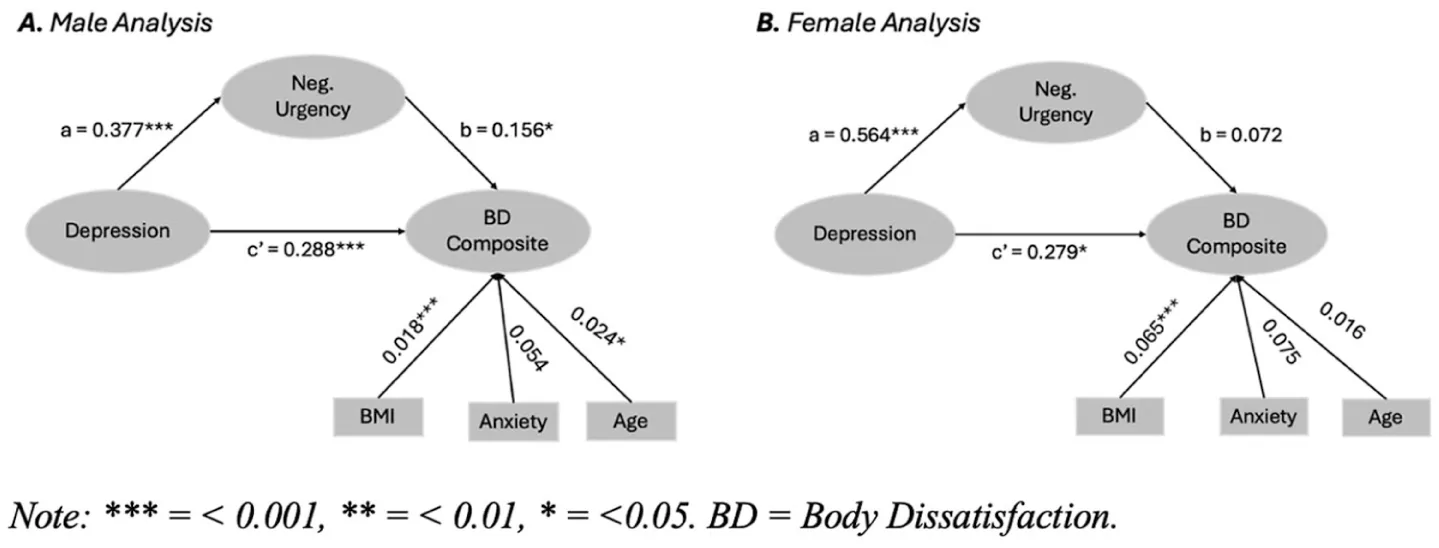
Sex-specific composite mediation model.

Among females (*n* = 105), the hypothesized mediation pattern was not supported (Figure 2, Panel B). Depression remained significantly associated with negative urgency (*a* = 0.564, *p* < 0.001); however, negative urgency did not significantly predict body dissatisfaction (*b* = 0.072, *p* = 0.058). The direct effect of depression on body dissatisfaction was significant (*c*’ = 0.279, *p* < 0.05). The model explained 12.63% (*p* = 0.046) of the variance in body dissatisfaction, consistent with small-to-moderate effects observed in psychosocial models, with BMI as a significant covariate (*p* < 0.001).

### Supplemental analyses: Shape concern and weight concern by sex group

#### Shape concern

Among males (*n* = 127), the mediation model examining Shape Concern showed significant effects (Figure 3, Panel A). Depression was significantly associated with negative urgency (*a* = 0.377, *p* < 0.001) and negative urgency significantly predicted Shape Concern (*b* = 0.165, *p* < 0.05). The direct effect of depression on Shape Concern remained significant (*c*’ = 0.283, *p* < 0.001), indicating partial mediation. The model explained 17.95% of the variance in Shape Concern (*p* = 0.042), indicating a modest explanatory contribution of affective and impulsivity-related factors, with BMI as a significant covariate (*p* < 0.001).

**Figure 3.**
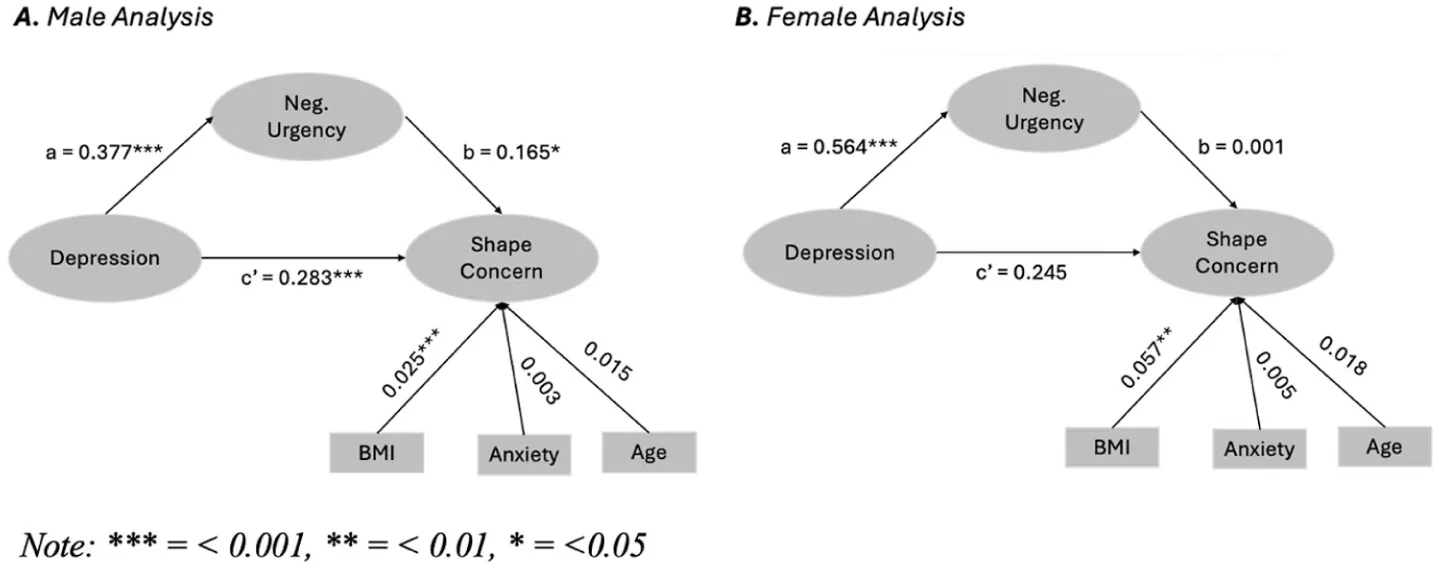
Sex-specific shape concern mediation models.

Among females (*n* = 105), the mediation model was not supported (Figure 3, Panel B). Depression was significantly associated with negative urgency (*a* = 0.564, *p* < 0.001), but negative urgency did not significantly predict Shape Concern (*b* = 0.001, *p* = 0.477). The direct effect of depression was also non-significant (*c*’ = 0.245, *p* = 0.582). The model explained 20.09% of the variance in Shape Concern (*p* = 0.029) reflecting a moderate effect size for the included predictors, with BMI as a significant covariate (*p* < 0.001).

#### Weight concern

For males (*n* = 127), the mediation model for Weight Concern also yielded significant findings (Figure 4, Panel A). Depression was associated with negative urgency (*a* = 0.377, *p* < 0.001), and negative urgency significantly predicted Weight Concern (*b* = 0.148, *p* < 0.05). The direct effect of depression on Weight Concern was significant (*c*′ = 0.292, *p* < 0.001), indicating partial mediation. The model explained 16.01% of the variance (*p* = 0.036), showing moderate power, with BMI as a significant covariate (*p* < 0.001).

**Figure 4.**
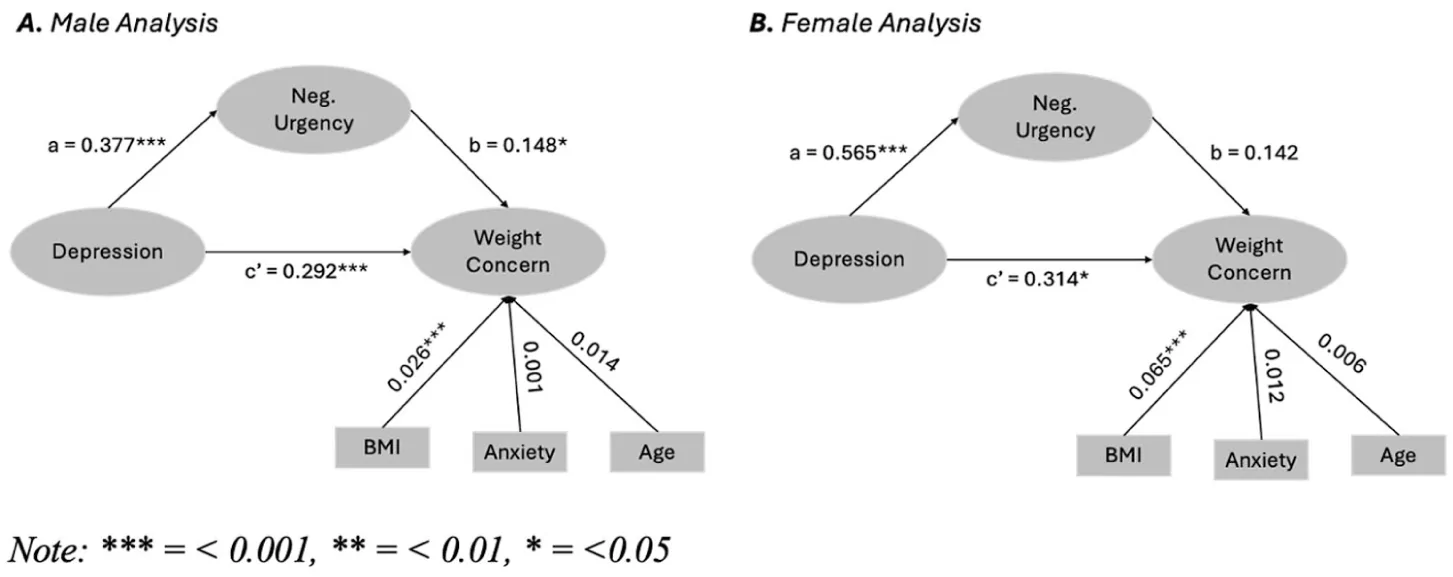
Sex-specific weight concern mediation models.

Among females (*n* = 105), the mediation pattern was not supported (Figure 4, Panel B). Although depression was significantly associated with negative urgency (*a* = 0.565, *p* < 0.001), negative urgency did not significantly predict Weight Concern (*b* = 0.142, *p* = 0.058). The direct effect of depression was significant (*c*’ = 0.314, *p* < 0.05). The model explained 20.34% of the variance in Weight Concern (*p* = 0.033), indicating moderate explanatory power within a cross-sectional psychosocial framework, with BMI as a significant covariate (*p* < 0.001).

## Discussion

This study investigated the role of negative urgency in body dissatisfaction, while modeling the contributions of state depression, state anxiety, BMI, age, and biological sex within a community sample of individuals aged 18–30 years. Given the cross-sectional design, mediation effects should be interpreted as associative rather than causal. The findings clarified the role of negative urgency in body dissatisfaction and underscore the importance of sex-specific mechanisms during this critical developmental window. Notably, while negative urgency emerged as a mediator in the relationship between depression and body dissatisfaction in the full sample, sex-specific analyses revealed this pattern was observed for males, but not for females. This sex difference was consistent across the body dissatisfaction composite, as well as the Shape Concern and Weight Concern subscales in specificity analyses, suggesting a robust pattern across multiple measures. Additionally, depression was consistently and strongly associated with negative urgency across both sexes, reinforcing its potential role as a dimensional psychopathology risk factor for impulsivity-related behaviors, consistent with prior literature (Whiteside and Lynam, 2001). Together, these findings emphasize the complexity of body dissatisfaction in young adults and the necessity of sex-informed approaches when designing interventions for this populations.

The most striking finding of this study is the clear sex difference in mediation patterns. In male participants, negative urgency consistently mediated the relationship between depression and body dissatisfaction across all measures, including the composite score, Shape Concern, and Weight Concern. This suggests that, among young men, negative urgency may represent a plausible pathway linking depressive symptoms to body dissatisfaction. However, this pattern was not observed in females. This sex difference aligns with evidence that males are more likely than females to express distress through behavioral means, such as impulsive actions (Chaplin and Aldao, 2013). Theoretical models further suggest that men tend to cope with emotional distress using externalized, action-oriented strategies rather than internalized rumination or passive withdrawal (Nolen-Hoeksema and Aldao, 2011). In practice, this may involve regulating negative emotions through impulsive or risky behaviors, including maladaptive actions directed toward the body—such as excessive exercise, disordered eating, or substance use (Nolen-Hoeksema, 2012). Within the context of body image, such patterns may manifest as impulsive attempts to alter appearance or heightened reactivity to perceived physique-related shortcomings, for example, using appearance- or performance-enhancing drugs in response to dissatisfaction (Hilkens et al., 2021).

In contrast, for female participants, negative urgency did not mediate the relationship between depression and body dissatisfaction, despite strong associations between depression and negative urgency in this group. This suggests that while young women experiencing depression also show increased impulsivity in response to negative emotions, this impulsivity did not translate into body dissatisfaction in the same way it did for males. Instead, other mechanisms may be more relevant for understanding body dissatisfaction in young women, such as internalized sociocultural pressures and stigma related to weight, social comparison processes, or cognitive factors like increased negative self-talk and rumination (Rodgers and Melioli, 2016; Selby et al., 2015).

These findings have important implications for understanding and addressing body dissatisfaction during young adulthood. The observed sex differences in the underlying mechanisms suggest that interventions may need to be tailored to the distinct psychological pathways driving body image concerns in males and females. In males, where negative urgency emerged as a mediator linking depressive symptoms to body dissatisfaction, interventions that directly address negative urgency—such as Dialectical Behavior Therapy (DBT), which emphasizes distress tolerance and emotion regulation skills—may be particularly effective for males, consistent with the observed mediation pattern (Linehan, 1993). Similarly, mindfulness-based interventions that enhance awareness of emotional states and reduce reactivity may attenuate distress-driven impulsivity (Chambers et al., 2009). Together, these results support future research examining whether interventions targeting negative urgency can reduce body dissatisfaction among young men.

Despite the lack of mediation findings in females, this group consistently reported higher levels of body dissatisfaction across all measures, a finding common in the literature (Groesz et al., 2002; Merino et al., 2024; Neumark-Sztainer et al., 2006). This underscores the continued vulnerability of young women to appearance-related concerns. For this group, interventions might focus more on addressing sociocultural influences, internalized appearance ideals, and depression itself, particularly how self-concept becomes impaired in depression. Evidence-based programs that incorporate media literacy, body acceptance, and cognitive restructuring strategies have demonstrated effectiveness in reducing body dissatisfaction among young women (Stice et al., 2007). Additionally, meta-analytic findings support the efficacy of stand-alone interventions that promote positive body image through self-compassion, cognitive reframing, and body functionality appreciation (Alleva et al., 2015). Taken together, our findings underscore the importance of sex-specific, developmentally appropriate interventions that consider the distinct mechanisms contributing to body dissatisfaction in young adults.

Beyond negative urgency, both higher BMI and higher depressive symptoms strongly predicted greater scores on the body dissatisfaction composite, as well as shape and weight concerns subscales in the models. These findings are aligned with a robust literature linking BMI to heightened exposure to weight-related stigma and internalized appearance norms (Ganem et al., 2009; Paxton et al., 2006; Penkal and Kurdek, 2007), and depressive symptoms to cognitive distortions and negative self-evaluation (Goldschmidt et al., 2014; Soares Filho et al., 2020). The dual presence of elevated BMI and mood disturbance may exert synergistic effects on body dissatisfaction, suggesting that integrative interventions—addressing both emotional well-being and body acceptance—may be especially effective in high-risk individuals. In contrast, anxiety was not a significant predictor of body dissatisfaction in any of the models. While this may be surprising given literature relating anxiety to body dissatisfaction (Meland et al., 2007; Vannucci and Ohannessian, 2018), it is possible the variance that attributed to anxiety could have been better explained by negative urgency, which is supported by literature on the more general relationship between these dimensions (King et al., 2022).

Despite the strengths of this study—including a diverse adult sample and rigorous multivariate analyses—several limitations should be noted. The cross-sectional design precludes causal inferences; longitudinal research is needed to establish temporal relationships between negative urgency, depression, BMI, age, and body dissatisfaction. Additionally, while this study focused on biological sex, future research should incorporate gender identity and consider the unique experiences of gender-diverse populations, who often face elevated risk for body image disturbances. Moreover, sociocultural and intersectional variables (e.g. race, ethnicity, SES) should be explored in future studies with more diverse samples to deepen understanding of body dissatisfaction and disparities therein. Finally, while this study focused on biological sex, future research should incorporate gender identity and consider the unique experiences of gender-diverse populations.

In conclusion, the present findings clarify the complex combination of predictors of body dissatisfaction. These findings are most applicable to community-dwelling young adults in high-income settings.. This study provides important new insights into the mechanisms underlying body dissatisfaction in young adults. Crucially, analyses revealed important sex differences in how negative urgency mediates the relationship between depression and body dissatisfaction, with this mediation pathway emerging in males but not in females. This specificity suggests that the mechanisms driving body dissatisfaction may differ by sex, underscoring the need for tailored prevention and intervention strategies. In sum, these findings indicate that body dissatisfaction in young men and women may emerge through distinct mechanisms, reinforcing the need to account for sex differences in both research and clinical practice. By elucidating these pathways, future work can guide the development of precise, sex-specific interventions that more effectively mitigate body image concerns and their associated risks during young adulthood. Addressing these nuances is essential to advancing prevention and treatment efforts in this critical developmental period.

## Data Availability

The data supporting the findings of this study are publicly available via the NKI-Rockland Sample: https://fcon_1000.projects.nitrc.org/indi/enhanced/.
